# Detection and isolation of wormhole nodes in wireless ad hoc networks based on post-wormhole actions

**DOI:** 10.1038/s41598-024-53938-9

**Published:** 2024-02-10

**Authors:** David Samuel Bhatti, Shahzad Saleem, Azhar Imran, Hyeon Jeong Kim, Ki-Il Kim, Kyu-Chul Lee

**Affiliations:** 1 Faculty of Information Technology, University of Central Punjab, Lahore, Pakistan; 2 Department of Cybersecurity, College of Computer Science and Engineering, Jeddah University, Jeddah, Saudi Arabia; 3 School of Electrical Engineering and Computer Science, National University of Sciences and Technology, Islamabad, Pakistan; 4 Department of Creative Technologies, Faculty of Computing & A.I., Air University, Islamabad, 42000 Pakistan; 5 Department of Computer Science and Engineering, Chungnam National University, Daejeon, 34134 Korea

**Keywords:** MANET attacks, Wormhole detection and isolation, Messaging overhead, Memory and processing costs, Detection rate, Packet loss and delivery ratio, Matthews correlation coefficient, Computer science, Information technology

## Abstract

The wormhole attack is one of the most treacherous attacks projected at the routing layer that can bypass cryptographic measures and derail the entire communication network. It is too difficult to prevent a priori; all the possible countermeasures are either too expensive or ineffective. Indeed, literature solutions either require expensive hardware (typically UWB or secure GPS transceivers) or pose specific constraints to the adversarial behavior (doing or not doing a suspicious action). The proposed solution belongs to the second category because the adversary is assumed to have done one or more known suspicious actions. In this solution, we adopt a heuristic approach to detect wormholes in ad hoc networks based on the detection of their illicit behaviors. Wormhole and post wormhole attacks are often confused in literature; that’s why we clearly state that our methodology does not provide a defence against wormholes, but rather against the actions that an adversary does after the wormhole, such as packet dropping, tampering with TTL, replaying and looping, etc. In terms of contributions, the proposed solution addresses the knock-out capability of attackers that is less targeted by the researcher’s community. In addition, it neither requires any additional hardware nor a change in it; instead, it is compatible with the existing network stack. The idea is simulated in ns2.30, and the average detection rate of the proposed solution is found to be 98-99%. The theoretical time to detect a wormhole node lies between 0.07-0.71 seconds. But, from the simulation, the average detection and isolation time is 0.67 seconds. In term of packet loss, the proposed solution has a relatively overhead of $\approx$ 22%. It works well in static and mobile scenarios, but the frame losses are higher in mobile scenarios as compared to static ones. The computational complexity of the solution is *O(n)*. Simulation results advocate that the solution is effective in terms of memory, processing, bandwidth, and energy cost. The solution is validated using statistical parameters such as Accuracy, Precision, F1-Score and Matthews correlation coefficient ($M_{cc}$).

## Introduction

Wireless ad hoc networks are infrastructure-less, self-configured, dynamic and have no centralized control of existing. They are simple, flexible and can be easily deployed in all environments. They can be seen working inside the body parts of living and non-living objects; for example, airborne wireless sensor networks (Airborne WSNs) for airplane monitoring system (AMS), smartphone ad hoc network (SPAN), wireless wearable body area networks are few examples of more advanced implementations of these networks^1–4^. Moreover, Internet of Things, Internet of Vehicles, mobile cloud computing, ad hoc clouds over the mobile ad hoc networks, and air traffic control systems are the hot emerging research areas of wireless ad hoc networks^5–10^. Ad hoc networks are working for the military to communicate efficiently, considering confidentiality requirements critical for its operations^11^. These networks are used to reinforce the battlefield when the nations are at war^12^. Such extensive use of ad hoc networks in military operations is giving rise to a new field of networking called the Internet of Battlefield (IoBT)^13^. SDN based ad hoc networks for military purposes are also being researched^14,15^. PANs, VANETs, Military or Tactical MANETs, VANETs, FLANETs, Navy Ad hoc Networks, Ad hoc Network of Robots, Disaster Rescue Ad hoc Network, Hospital Ad hoc Network, BANs etc. are the different applications of wireless ad hoc networks^16–19^. Considering the sensitivity of ad hoc networks, it is evident that security is of paramount importance and must be addressed as a top priority due to the broadcast nature (open medium) characteristic of such networks. This kind of medium is significantly susceptible to various attacks directed at multiple layers of the TCP/IP model. Among these attacks, the wormhole attack stands out as one of the most challenging and inherently hard to detect before it occurs. It is particularly disruptive during the routing process in shortest path routing protocols such as AODV (Ad Hoc On-demand Distance Vector), and DSR (Dynamic Source Routing). In MANETs, routing protocols are categorized into three types, Reactive (e.g AODV^20^, DSR^21^), Proactive (DSDV^22^, OLSR^23^ (Optimized Link State Routing)) and Hybrid (ZRP^24^ (Zone Routing Protocol))^25–27^. Proactive routing protocols are suitable for low mobility and relatively higher data rates, reactive protocols good for high node mobility and relatively lower data rates, whereas hybrid protocols are balanced between these two types^28,29^. DSR is a source routing protocol that allows the source node to determine the entire route to the destination. In contrast, AODV is a destination routing protocol, which establishes a route through a sequence of hops from one node to the next until reaching the intended destination. In high mobility scenarios, AODV out performs DSR, owing to the higher probability of link breaks in DSR due to its reliance on source routing, while AODV, with its table-driven routing, encounters fewer disruptions. Since, our proposed solution is based on AODV that’s why we would like to discuss it briefly in this section.

**Figure 1:**
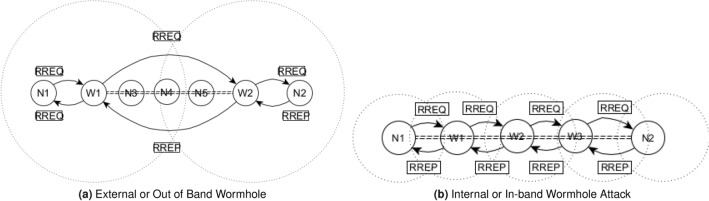
Wormhole.

AODV is reactive, self-configuring, and loop-free routing protocol designed for wireless node communication. It effectively handles issues related to mobility, link failures, and packet losses. Its routing table contains essential fields: next-hop, sequence number, and hop-count. The sequence number signifies route freshness, hop-count gauges distance, and the next-hop indicates the subsequent node in the route. In a scenario involving nodes A, B, and D (with D as the destination), if B serves as A’s next hop to D, AODV adheres to the following property: (($Seq_A < Seq_B$) OR ($Seq_A = Seq_B$)) AND ($HopCount_A > HopCount_B$). This ensures that B possesses a newer or shorter route to D in comparison to A, as detailed by Perkins et al.^20^. The wormhole attack exploits this inherent property of shortest routes for its projection.The bigger problem of this attack is its mounting with the help of two or more colluding nodes. In this attack, attackers create a private high radio-range link and allure the traffic from their neighbors while giving them an illusion of a shortest path. Usually this illusion is of two hops. So, RREQ originated by the source nodes reaches its destination earlier through the wormhole link and RREP in response to RREQ reaches source using the same route. In AODV, forward and backward routes are symmetric. Wormhole node near the source captures data packets and passes them to the other attacker node. This attacking node can replay, broadcast, and drop these packets. In addition to this, these two colluding nodes can put these packets in loop causing them to drop^30,31^. Such wormholes are called out-of-band wormholes, which are easier to detect and isolate as compared with in-band wormholes that are very difficult to detect due to using the same radio link. In-band wormholes project using encapsulation, packet relay and protocol deviation methods^32^. They are treacherous and tough to detect at launching or wormhole creation time. If they remain silent, they remain undetected but if they act maliciously after wormhole establishment then can be caught and isolated. It is worth mentioning that both grayhole and blackhole attacks are types of single-node attacks. A grayhole attack is a partial denial-of-service attack, where a malicious node selectively drops or modifies a subset of data packets. In contrast, a blackhole attack is a complete denial-of-service attack, with the malicious node intercepting and discarding all data packets passing through it. Unlike grayhole and blackhole attacks, a wormhole attack involves collusion between two or more malicious nodes working together to create a tunnel or “wormhole” between them. Key characteristics of a wormhole attack include tunnel creation, packet redirection, disruption of network topology, and the potential for security threats such as replay attacks. Present solution detects nodes colluding with one another, blackhole, but not addresses the grayhole in particular. So, it clearly, states that if wormhole perform grayhole type of actions even after wormhole creation it becomes hard to detect them. But, the probability of such wormhole establishment is low. It is also noticed, they attract small traffic that corresponds to low impact on the overall network performance^33,34^. Furthermore, These two types of wormholes are shown in Fig. 1. In Fig. 1a, W1 and W2 are with the help a private high radio-range link create out-of-band wormhole allure maximum the traffic from its surrounding. In Fig. 1b, W1, W2, and W3, are forming in-band wormhole using common radio link. The basic taxonomy of wormhole attacks in terms of classification is open, closed and half-open. Some time, they are also classified on the basis of attack vector such as packet encapsulation, and relay, high transmission power, high quality link or protocol deviation. Valuable insights concerning them can be explored from the in-depth research presented in^35,36^, providing a comprehensive understanding of these classes.

In this article wormhole means adversaries successfully create a link by giving illusion that this a shortest route. Post-Wormhole means that now the attacker can project different malicious attacks such eavesdrop on data, inject malicious traffic, traffic analysis, drop or modify packets, and potentially launch further attacks.

From the related work, it is observed that most of the previous techniques need additional hardware or require a change that may compromise the scalability of the network^37^. Wormhole can bypass cryptographic measures, so the techniques based on complex computing functions are less welcomed in low-resource scenarios of ad hoc networks. That’s why devising solutions for these scenarios is one of the critical challenges^38^.

The goal of this study is to develop a cost-effective solution in terms of processing, storage, and messaging (bandwidth) that could detect and mitigate the wormhole attack without incurring the extra overhead of hardware (e.g. directional or GPS antenna, clock synchronization, additional nodes) or making a change to it. Since in hostile environments, it is difficult to replace/recharge the batteries of wireless devices, the installation of energy-aware protocols ensures a long life of these devices. In the proposed approach, legitimate nodes maintain a history of neighbours’ behaviour, which is used to detect the suspicious and malicious nature of the wireless node. Upon receiving a reasonable number of reports from neighbours about the suspiciousness of a node, the reported node is announced as malicious and placed in the block list. The legal nodes refresh their routing tables, and no route request (RREQ) from these malicious nodes is forwarded in the future. Unfortunately, if such an RREQ is forwarded, it may create a wormhole link. The proposed solution is tested via ns-2.30 with varying degrees of wormhole length and the number of attacker nodes, with static and mobile nodes moving at different speed rates and varying thresholds of suspicious and malicious alerts. Simulation results show that our solution successfully detects and isolates the wormhole nodes. Rest of the paper is organized into seven different sections, namely; 2 Related Work, 3 Wormhole Attack Scenarios, 4 Proposed Solution, 5 Detection Time Analysis, 6 Overhead Analysis, 7 Limitations and Research Challenges, 8 Conclusion and Future Work. Furthermore, an abbreviation DAIWN is used to refer proposed solution in this manuscript.

### Contributions

The salient contributions of our study are given below.

1. Proposed approach successfully detects and isolates wormhole nodes with a high rate that is 98-99% and enables the network to recover within a small period of 0.151–1.186 seconds.
2. It does not require additional hardware, such as GPS, guard nodes, tight/loose clock synchronization, etc., that would limit the system’s potential to scale and be cost-effective. But, a small amount of additional storage space would be required to store, suspicious/malicious alerts and nodes.
3. The solution is compatible with the existing network stack (TCP/IP). It means making a small change in the software of a routing protocol, the proposed solution can be accommodated in the existing technology. Moreover, we do not need to make any hardware changes in devices.
4. It has been validated and simulated in NS2 and satisfies the statistical benchmarks for accuracy, precision, F1-Score, and Matthews Correlation Coefficient (MCC). The solution is adaptable and is simple to upgrade to counter new attack vectors.

### Assumption

1. The attackers are not considered smart in other literature, so to be prudent and vigilant with the proposed alert mechanism, we assume the attackers might be too smart to knock out the legitimate nodes from the network by generating the same type of alerts. The mentioned assumption makes this study unique, as other solutions do not discuss this feature of an attacker.
2. We assume the wireless nodes are in promiscuous mode, which allows them to capture and process all network traffic passing through a specific segment, including packets not destined to their own MAC addresses. Promiscuous mode is useful in networks including ad hoc for network monitoring and analysis, security and intrusion detection, troubleshooting connectivity problems, diagnosing performance issues, and identifying security vulnerabilities etc. while privacy and legal considerations as well. It is worth to mention that not all device and driver support provide this feature but mostly do. There is significant number of articles that specifically propose the promiscuous mode wireless ad hoc networks for security, performance analysis, and diagnostic purposes such as^39–44^. Simultaneously, while promiscuous mode consumes extra energy by continuously monitoring the medium, the advantages it brings forth outweigh this drawback.

## Related work

An extensive literature survey is carried out on the detection and prevention of wormhole attacks for a better understanding of other researchers’ work and to contribute to the existing body of knowledge. Abdan et. al.^45^ proposed a Machine Learning based solution for the detection of wormhole attacks using a K-nearest neighbour), support vector machine, decision tree, linear discrimination analysis, naive Bayes, and convolutional neural network. According to their results, the decision tree performed better than others in terms of detection accuracy, which was 98.9%. Muhannad et al.^46^ recently proposed HWAD, a hybrid approach for the detection of wormhole attacks. They made a good use of two network performance parameters, RTT and packet delivery ratio, for detecting in-band wormhole nodes and transmission range for out-of-band wormhole nodes. Moreover, the authors of this research made use of the K-Means clustering algorithm, which is computationally efficient and is extensively used in networks for the detection of malicious behaviour of nodes. The solution proposed by the authors is simple and easy to deploy. It does not incur additional costs in terms of hardware, clock synchronization, or complex cryptography; instead, the scheme makes use of advanced machine-learning technology for optimization and accuracy.

Kamaleshwar et. al.^47^ proposed a self-adaptive framework for the detection and isolation of wormhole and black-hole attacks in 6 Low-power Wireless Personal Area Networks (6LoWPAN). They used to send dummy RREQ packets for filtering out the shortest route (2-3 hops) and finding the average route (4-5 hops). They used watch nodes for observing the malicious behaviour of attacker nodes and assigned ranks to those nodes. On the basis of these ranks, authors detect and isolate malicious nodes. Their solution is simple that does not require extra resources. It can effectively detect wormhole nodes with a radio of high range. However, wormhole nodes with low radio range that establish normal routes may be left undetected. Shukla et. al.^48^ proposed an ECC-based (Elliptic Curve Cryptography) technique for the mitigation of wormhole and blackhole attacks. They modified the AODV protocol for the proof of concept and succeeded in saving energy. Such a technique works well for resource-enriched scenarios, but for low-powered devices such as wearable and WBANs etc., these solutions prove to be expensive due to complex cryptographic functions.

Tamilarasi et. al^49^ adopted a quite reasonable and optimized approach for selecting the route that is free from wormholes in WSNs. The selection is based on the feedback mechanism in which the source sends a Detection-Packet (DP) on multiple routes. In response to DP, Feedback-Packet (FP) is sent back on the same route by the destination. From the comparison and analysis of RTTs and Hop-Counts of both the packets, it is decided whether a route is free from wormhole or not. After this initial selection, the Particle Swarm Optimization (PSO) algorithm is used to select the energy-optimized route. The proposed technique is simple and flexible. However, PSO is a repetitive computational process and hence we argue that it can quickly drain the battery of a sensor. We shall refer to this research as DWASPS in subsequent discussions. Ohida et. al.^50^ proposed one of the simplest protocols for wormhole detection. It is based on the time taken by the frame to travel from source to destination. A frame that traverses the wormhole link will have a shorter time as compared to a link that is free from the wormhole, provided the number of hops remains the same in both cases. Instead of a single-hop communication between the base station and the WSN nodes, it is considered a multi-hop in this research work. Ohida et. al.^50^ protocol is an extension of classic time-based leashes used to detect wormhole nodes^51^. We will refer to this protocol as MCRP in later discussions. One drawback is that the solution requires clock synchronization which only works on more advanced and sophisticated hardware^52^.

Xiao Luo et. al.^53^ proposed a very simple and localized protocol (CREDND) against wormhole detection. It does not require any extra hardware, clock synchronization, or nodes’ coordinates information. CREDND has the capability to detect and isolate both internal (wormhole nodes similar to normal ones behaving abnormally) and external (out-of-band attack) wormholes. Their solution is based on hop counts and the trust of legal nodes in the neighbours.

Qazi et. al.^54^ proposed multi-rate DelPHI (mDelPHI), which is an extension of classic DelPHI^55^. The authors proposed a solution for wormhole mitigation in multi-rate wireless scenarios because classical DelPHI does not take this factor into account. The mDelPHI algorithm is installed on each node that is supposed to calculate the base-band rate of every hop transmission, packet processing time, queuing delay and the time taken to allocate a channel for a particular transmission. Each node watches the activities of its surrounding nodes; any delay in forwarding the packet that is greater than the threshold may be an indication of the existence of wormhole nodes. For instance, the RTT of two malicious nodes or a wormhole tunnel would be very low or much higher as compared to the RTT between two normal nodes. mDelPHI is a simple technique that does not require any additional hardware, cryptography, clock synchronizations, or any other form of extra hardware. Narayanan et. al.^56^ proposed a modified version of AODV for the prevention of wormhole attacks. They used two parameters RTT and PFR $\left[ PFR=\frac{PacketsSent~(Sender)}{PacketsReceived~(Receiver)}\right]$ for the detection of a malicious node. PFR (Packet Forward  Ratio) less than one and RTT less than the pre-calculated threshold determine the existence of wormhole nodes in the network. This technique is simple and free from the extra cost of additional resources. The practical implementation of the wormhole attack is shown by Pericle et. al.^57^ on 6LoWPAN network by using RPL (Routing Protocol for Low-Power and Lossy Networks). They actually tested the realization of wormhole attacks on Wireless Sensor and Actuator Networks (WSANs) and studied their impact on these networks. They also tested different solutions proposed in the literature in order to test their suitability. They proposed to first let the wormhole attack succeed and then counter the subsequent malicious activities of the attack.

A trust-based approach is proposed by Gupta et. al.^58^. In this study, the behaviour of wireless nodes is observed and Faith-Value is calculated. The nodes communicate this value with each other. The behavior of a receiving node is challenged every time a node from the network wants to send a message to it. This calculated faith value is renewed with the behavior drawn from the sent and received packets by that particular node. The contents of a message are encrypted using a credibility-based cryptographic function. The encrypted message works when appended with the signature and faith value of the respective node. This technique is capable of detecting wormholes, black holes, and even jellyfish attacks in delay-tolerant networks. Sharma et. al. the authors of^59^ found a high-transmission-power related wormhole vulnerability in AODV based WSNs. In this technique, a modified AODV monitors every sensor node, and if the protocol finds any RREQ from the route where node transmission power is higher than the threshold, then it is discarded and the route is placed in the black list. In future, none of the RREQ is accepted from the list of routes that are already listed in the blacklist. It is a simple approach that does not require additional hardware, clock synchronization etc. But, it detects only high transmission-power based wormhole nodes which is one of the concerns for the practicality of this solution. Detecting the wormhole nodes by using the concept of Honey-Pot is one of the smart strategies. Honey-pots allure attackers toward themselves which helps to detect the malicious behavior of attacker nodes easily. SIPHON^60^ is one such research that suggests the use of honey-pots for the detection of wormhole attacks. Wormhole vulnerability in 3G/4G networks is analyzed by Guangdong Bai and Zhang Qing in^61^. They worked in real wormhole-vulnerable scenarios. They scanned more than 16 million mobile users. These users belong to three main internet service providers in China. These are China Mobile, China Telecom and China Unicom. About 2% of these users were found using applications that were installed with a wormhole vulnerability. The authors of this research also used a simple honey-pot-based methodology for the detection of the wormhole vulnerability. The honey-pot-based techniques are simple, but require some additional devices to be installed in the form of honey-pots. Reza et al. proposed a technique named DAWA in their interesting work^62^. The human immune system was exploited with the help of artificial intelligence for the detection and isolation of wormhole nodes in mobile ad hoc networks. The approach is novel, but it requires intensive computing power due to the use of AI algorithms that need to be trained efficiently for proper detection and isolation of wormhole nodes. It does not suit resource-constrained wireless scenarios because it exercises a lot of complex mathematical and statistical operations.

Gul-Hsin Lai^63^ proposed a rank-based approach for detecting and removing wormhole nodes from WSNs. The author exploited the rank field of RPL (Routing Protocol for Low-Power and Lossy Networks) messages such as DIO and DAO to detect malicious nodes. RPL is the standard routing protocol designed for 6LoWWPAN. The authors of this study used hop count to calculate the rank threshold, which is the difference between the parent rank and the node itself. It is calculated at the beginning of a network, assuming there is no wormhole. Rank-difference is the same calculation as stated above but performed at another time. If the rank difference obtained from the DIO message of RPL is greater than a threshold, then the DIO message’s sender is treated as a malicious node. It is a simple technique that does not add any hardware cost and does not use any complex cryptographic functions. But the solution assumes that there is no wormhole node at the beginning of a network, which is not always true. Rupinder Singh et al. proposed WRHT^31^, in which a watchdog^64^ and DElPHI (Delay Per Hop Indicator)^55^ based approaches were combined. WRHT is a probabilistic approach that calculates the probability of the existence of a wormhole in the established path using the probability factor of delay time and packets lost. The scheme is simple and handles almost all types of attacks, but it suffers from the limitations of probability theory.

Shiyu Ji et al.^65^ proposed a network codding-based approach for detecting wormhole attacks. This is one of the simple techniques that does not require any extra hardware, clock synchronization, cryptographic functions, or GPS. It operates on local information only. This way, it does not add any extra messaging cost to the communication system. Shivangi Dwivedi and Priyanka Tripathi^66^ have suggested a simple approach in which the source and destination exchange their neighbor lists. A wormhole announcement message is sent if the count of common neighbours is greater than the set threshold. Upon receiving the message, the legal nodes drop the wormhole nodes from their neighbour list. Luo et. al.^33^ proposed a wormhole detection technique based on the concept that a large amount of traffic is attracted by a wormhole link. But, for in-band wormholes, the proposed approach does not perform well because in-band wormhole attackers use the same normal radio link and thus does not allure too much traffic toward itself resulting in the low detection probability. Unit disk graphs provide graph-theoretic models for broadcast networks like wireless sensors, ad hoc, and cellular networks. Unit disk graph is the intersection area of the circles having alike size^67^. These models were used by Rakesh et al. in^68^ for detecting and isolating wormhole nodes from ad hoc networks. This technique is simple and free from extra hardware, clock synchronization and complex cryptographic operations.

Zubair et al.^69^ proposed a technique for wormhole detection, that is based on the sharing of routing table information among the network nodes. It is an efficient technique for long radio-range attackers. E2SIW^70^ is based upon a shorter distance between nodes which are involved in creating a route from source to destination. It is an improvement of De-Worm^71^, but E2SIW has reduced control messaging overhead compared to De-Worm. De-Worm also used GPS to mitigate wormhole attacks, which adds additional hardware cost, but E2SIW does not do this.

Ming-Yang Su proposed WARP^52^. It is based on the principle of anomaly detection. It adopts an approach where no extra hardware is required, no loose or tight time synchronization is needed, and it is free from heavy computations of cryptographic functions. In this sense, it is a low-cost solution. WARP is more suitable for scenarios where the attackers have a high radio range as compared to ones where there are normal radio range attackers.

MOBIWORP^72^ is a good approach for the mitigation of wormhole attack. It suits well for resourceful networks. It uses public key infrastructures where a certification authority (CA) has more storage capacity and processing capabilities as compared to the other normal nodes. MOBIWORP requires high bandwidth and processing power for signature verification and authentication. LITEWORP^73^ uses clock synchronization. It has a good rate (98.9%) of filtering non-malicious routes from malicious ones. The rate of missed detection increases with network density. LITEWORP uses a combination of one-time authenticated neighbor discovery protocol along with guard nodes that attest transmission. The neighbor discovery process can be vulnerable to wormhole attack if the attack is launched prior to such discovery. LITEWORP makes use of clock synchronization and precise synchronization needs hardware implementation as reported by^52^.

There are some comprehensive studies on reviewing different features for the detection and isolation of wormhole attack like^74,75^. One can use Table 1 for a quick review and understanding of these techniques.

### Conclusion of the literature review

1. The literature review shows that most mitigation techniques are based on additional hardware, complex cryptography functions, loose or tight clock synchronization, GPS, RTT, hop counts, graph theory, packet leashes, etc.
2. It is also concluded that the scalability of an ad hoc network is reduced by using clock synchronization and extra hardware (e.g. GPS)^37^.
3. Cryptographic measures fail to deal with wormhole attacks. So, a light, simple solution free from the complexity of cryptographic functions, extra hardware, clock synchronization etc., is needed to deal with this attack.
4. Furthermore, if wormhole attackers opt for regular links rather than high-quality ones, they could evade detection during the wormhole formation. Subsequently, if they engage in disruptive “grayhole” actions post-wormhole establishment, still their detection remains a challenge. It’s important to note that the likelihood of such wormhole creation is minimal. Additionally, these attackers tend to attract minimal traffic, resulting in limited impact on the overall network performance^33,34^.

**Table 1:** SUMMARY OF WORMHOLE MITIGATION TECHNIQUES.

Method	Requirement	Commentary
Abdan et. al.^45^	Training, Learning, Data Set	ML solutions are computationally costly for low-powered devices
HWAD^46^	RTT, packet delivery ratio with K-Means Clustering	handles in-band and out of band wormholes
Kamaleshwar et. al.^47^	rank based approach, does not require extra hardware	may miss low or normal range wormholes
Shukla et. al.^48^	cryptographic-based approach	not suitable for low-resource environments
DWASPS^49^	RTT and hop-count anomalies are exploited	Particle Swarm Optimization affects energy of a sender
MCRP^50^	Packet traveling needs multi-hop wireless network	Based on packet leashes, so requires time synchronization
WARP^52^	WARP is based on anomaly detection, avoid routes with nodes having high capacity of building routes	good solution for ad hoc networks with slightly low delivery performance; it has high packet loss ratio
Xiao Luo et. al.^53^	no extra demand of hardware, GPS, time synchronization	Trust-based solution which is based on hop-counts
mDelPHI^54^	RTT, packet processing time and queue delays are used	mDelPHI is an extension of DelPHI. It covers multi-rate communication system without additional hardware
Pericle et. al.^57^	They proposed to let the wormhole occur and later protect the network from its malicious acts	practically implemented the wormhole on 6LoWPAN and tests the suitability of different proposed solution
Narayanan et.al^56^	RTT and packet-forwarding-ratio based approach	simple, free from additional hardware costs
Gupta et al.^58^	trust-based faith value is used to detect a malicious node	detects wormhole, blackhole and jellyfish
SIPHON^60^	honey-pots used	additional nodes are deployed in the form of honey-pots
DAWA^62^	novel, but requires intensive computing	human immune system is used with artificial intelligence
Kathrik et al.^76^	RTT-based, assumes all nodes have same radio range	fails over frequent connection changes and congestion
Sharma et al.^59^	uses transmission power	detects only high radio range attackers
Gul-Hsin Lai^63^	rank-based approach uses DIO and DAO of RPL protocol	assumes there is no wormhole at the beginning of a network
WRHT^31^	based on watchdog^64^ and DElPHI^55^	limitations of probability theory
Shiyu Ji et. al.^65^	extra hardware, clock sync, cryptography, GPS not required	network codding based approach, computationally complex
Rakesh et al. in^68^	uses unit disk graphs^67^	simple technique
Shivangi et. al.^66^	exchange of neighbor lists	in dense network it incurs high cost; it can be expensive in terms of bandwidth, storage, memory and energy
E2SIW^70^	Extension of De-Worm^71^ and based on shorter distance	E2SIW reduces messaging overhead
Zubair et. al.^69^	sharing of routing table information	suitable for long radio range attackers
MOBIWORP^72^	cryptographic concept with CA synchronization	not suitable for low-resource environments due to CA
LITEWORP^73^	clock synchronization, secure key sharing protocol	precise clock synchronization needs hardware implementation, neighbours discovery might be vulnerable to attack

## Wormhole attack scenarios

The shortest-path routing protocols (e.g AODV) are the potential victims of wormhole attacks. A wormhole projects itself at the time of the route discovery process with the help of two or more colluding nodes. In this attack, the radio range of the attackers is higher than the normal nodes. So, RREQ originated by the source node reaches its destination earlier via the wormhole link and the RREP message reaches the source using the same route. In AODV, forward and backward routes are symmetric. So, the wormhole node near the source captures data packets and passes them to the other attacker node, which can replay, broadcast, and drop these packets. In addition to this, the two colluding nodes can put packets in a loop and subsequently dropped^30,31^.

Wormhole nodes may have a private high radio-range link and allure the traffic from their neighbors while giving them an illusion of the shortest path; usually, this deception is of two hops. It is called “out-of-band wormhole” and is easier to detect and isolate. But for in-band wormholes, attackers have the same normal radio range as the legal nodes. They are slightly difficult to detect. The basic taxonomy of wormhole attacks in terms of classification is open, closed and half-open. Sometimes, they are also classified on the basis of attack vectors such as packet encapsulation, relay, high transmission power, high-quality link (out-of-band channel) or protocol deviation. We can study them further from^31,35,36^. The concept of a wormhole is discussed below with the help of two use cases they may help in deciding the thresholds of alerts.

**Figure 2:**
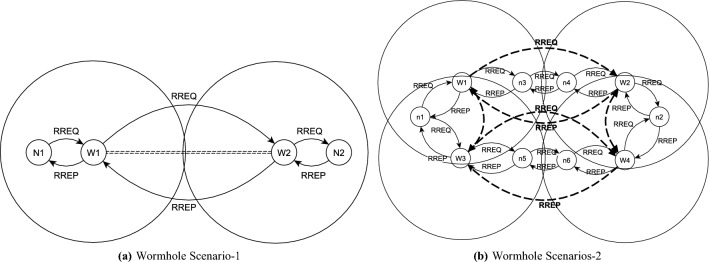
Wormhole Scenarios.

**Legal Nodes **$\le$** Wormhole Nodes** When the alerts threshold is set to 50%, the legitimate nodes can knock out malicious nodes successfully by generating suspicious/malicious alert messages. This means, if 50% of the network nodes detect malicious activity and report it to all the other nodes through the alerts, then the wormhole nodes can be detected and isolated. In this scenario, attackers can exploit the proposed security mechanism and isolate legal nodes because they are also equal to or greater than 50% of the network nodes as shown in Fig. 2a.

**Legal Nodes **>** Wormhole Nodes ** In this scenario, attackers cannot isolate legal nodes because they are less than 50% of the legal network nodes. Figure 2b shows that legal nodes can easily isolate wormhole nodes as they are more in number as compared to wormhole nodes.

From the discussion made in these scenarios, threshold of the network alert messages can be acquired using Eq. (1).

1 $$\begin{aligned} \small n = \left( N-1\right) * \frac{x}{100} \end{aligned}$$

n = number of malicious alerts from the network nodes

N = number of nodes in the network

x = the percentage

Equation (1) helps to efficiently detect long-range attackers. Therefore, a greater number of legitimate nodes have the chance to watch the activities of these attackers. But, it reduces when low-range malicious nodes come into the network; because due to low radio range very small number of legitimate nodes would be able to observe their activities. So, when this small number of nodes will report the malicious behaviour of the attacker nodes, then there is a high chance that the number of alerts the legitimate nodes generate may not reach the threshold. So, the proposed alert mechanism might fail to isolate a reasonable number of malicious nodes from the network. From this discussion, we can easily decide that by making a slight improvement in the above equation, the proposed solution will be able to detect the normal-range attackers as well. Equation (2) is the improved form of Eq. (1). Equation (2) helps compute the number of alerts that a node expects from its neighbours for the detection and isolation of low radio range attackers.

2 $$\begin{aligned} k=\left( N-1\right) *\frac{1}{100}*\left( \frac{y\pi R^2}{A}\right) \end{aligned}$$

where: k=number of malicious alerts from neighbours nodes, N=number of nodes in the network, R=radio range of the nodes, A=network size (area), y= the percentage.

By increasing the number of legal nodes in the network, the rate of detection and isolation of malicious nodes also increases. On the other hand, when the sender nodes in the network or connections reset increase, the attack probability also increases. So, in dense networks, the attack probability increases, the probability of detection and isolation of malicious nodes also increases but knocking-out of legal nodes becomes hard. From all these cases we have concluded that isolation is an interdependent function of the attacker and legal nodes in the network. So, if wormholes nodes gets equal or greater than legal nodes, there is a equal chance of knock out of legal nodes as well. We can say that the malicious node detection rate is a function of **m** and **k**, where **m** is the number of alerts excluding the alerts that are received from the neighbours of a receiver. It means there must be some alerts from other parts of the network excluding the neighbours to conclude that a particular node is bad. From Eqs. (1) and (2), we can derive **m**, as given in Eq. (3). It helps in finding the threshold to be set for the low radio range attackers.

3 $$\begin{aligned} m=\left( N-1\right) *\frac{1}{100}*\left[ x-\left( \frac{y\pi R^2}{A}\right) \right] \end{aligned}$$

Overall, we can say that $n\approxeq k+m$, if we want to set the threshold of alerts based upon the whole network.

The concept suggests that in the presence of numerous attacking nodes, legitimate nodes can be rendered isolated. This isolation is determined by a threshold set at 50%, indicating that if over 50% of neighboring nodes identify a node as malicious, it is isolated. Although the likelihood of a significant number of attackers in a network is low in practice, if such an event occurs, the probability of detection increases. However, in a scenario where the network comprises more than 50% attackers, there exists a possibility of its non-existence and subsequent collapse. Technically, proposed solution is not handling fake alerts, it is the threshold (50%) which is considered to avoid the isolation of legitimate nodes. Moreover, if one attacker is isolated, non-isolated is unable to carry out any malicious action independently, as the creation of a wormhole requires a companion.

## Proposed solution

We let the wormhole occur and later judge the post-wormhole operations of nodes. For this purpose, we assume the nodes are in promiscuous mode; they can listen to the activities of their neighbouring nodes, maintain a cache/list of these activities, analyze their cache/list, and from the analysis they can detect and isolate the malicious nodes from the network. A node; upon successful detection of suspicious activity of another node, generates a suspicious alert. Every node counts suspicious alerts against each reported node. A node that counts at least **n** suspicious alerts, generates a malicious alert. Where **n** is the number of suspicious alerts needed to announce that the node is a malicious member. When a node receives **k** number of malicious alerts from different nodes in the network, it will place the reported node in the bnList and no more packets will be entertained from this node. The notations used in the subsequent part of this manuscript are given in Table 2.

**Table 2:** Symbols Used.

Symbol/	Used for	Symbol/	Used for
Abbreviation		Abbreviation	
$\leftarrow$	Assignment	Msg	Message
$\in$	Belongs to	$\alpha$	to write 1-byte
$\notin$	Not belongs to	$\beta$	to read 1-byte
=	Equal to	$\gamma$	to send 1 bit
$\ne$	Not Equal to	$Mem_{Bytes}$	Memory Bytes
Pkt	Packet	$Msg_{Bytes}$	Message Bytes
sAlert	Suspicious Alert	$Mem_{Energy}$	Memory mJ
mAlert	Malicious Alert	$Msg_{Energy}$	Message uJ
Mal	Malicious	Prev	Previous
nxt	Next	Src	Source
Dst	Destination	BrdCast	Broadcast
snList	Suspicious Node List	bnList	Block Node List

To make the solution easy to understand, it is divided into two parts i) detection of wormhole nodes, ii) isolation of wormhole nodes. First, we shall discuss the detection algorithm and then isolation.

### Detection of malicious nodes

Algorithm 1 is used to detect a wormhole node. It is designed to address the following cases. These cases explain this algorithm very well. To detect the malicious behaviour of wormhole nodes, it makes use of a data structure similar to the one given in Table 3. These cases are discussed one by one. The data structure (pktList) is used to maintain and store packet information

**Case 1:** If the sniffing node sniffs a packet where the intermediate/relay node has changed the destination-IP to broadcast-IP, it will generate a suspicious alert. It can be seen from Table 3 that in the case of Packet-A there is an abnormal decrease in TTL, which ultimately causes packet drop.

**Case 2:** This is the case in which the intermediate malicious node swaps the next hop MAC-Address with the previous one or vice versa. This causes the data packet to remain in a constant loop until its TTL reaches zero and it is dropped. Table 3 shows that Packet-B is facing a loopback issue. In this case, legal nodes are being compromised and the behaviour will cause smart packet dropping.

**Case 3:** The intermediate malicious node decreases the TTL of a packet by more than a value of 1 and causes it to be dropped before reaching its destination. A malicious node may increase the TTL value of the packet, causing it to keep on moving in the network. This bad behaviour of a wormhole node creates energy drainage issues at legitimate nodes. Packet-C in Table 3 shows that legal nodes are compromised, and their energy can drop to zero in the case of continuous or high increment in the value of the TTL of the packet.

**Case 4:** In this case, the first malicious node captures packets from one part of the network and relays it to its companion node, which is located in another part. This is also a malicious node that replays the packets in this part of the same network. We have tried to show this behaviour using Packet-D in Table 3 where a legal node is also participating in loop creation causing smart packet drops. This scenario usually happens with normal radio range attackers.

**Case 5:** It can be seen from Table 3 that as soon as a legal node transmits a packet, it is captured by a wormhole (W2) node, which tunnels this packet to another wormhole node (W10), thus badly corrupting the TTL value of the packet along with replacing the original IPs with their own. The wormhole node (W10) tunnels this packet to the wormhole node (w2) resulting in no more forwarding of this packet. This can be seen from Packet-E given in Table 3 where loopback is created, causing packet drop. Usually, such behaviour is shown by high radio-range attackers. Even, if they do not corrupt the original IPs, still the packet will be trapped due to the high radio range of the wormhole nodes.

**Case 6:** This is a scenario where the malicious nodes replace the source-IP or destination-IP with companions or other nodes causing the packet to be in loops or reaching to undesired locations. Referring Table 3, Packet-F shows that data packets can be replayed and broadcast in this case. Such an attack targets network life, in other words, the attack is on the energy of the network nodes.

**Figure 3:**
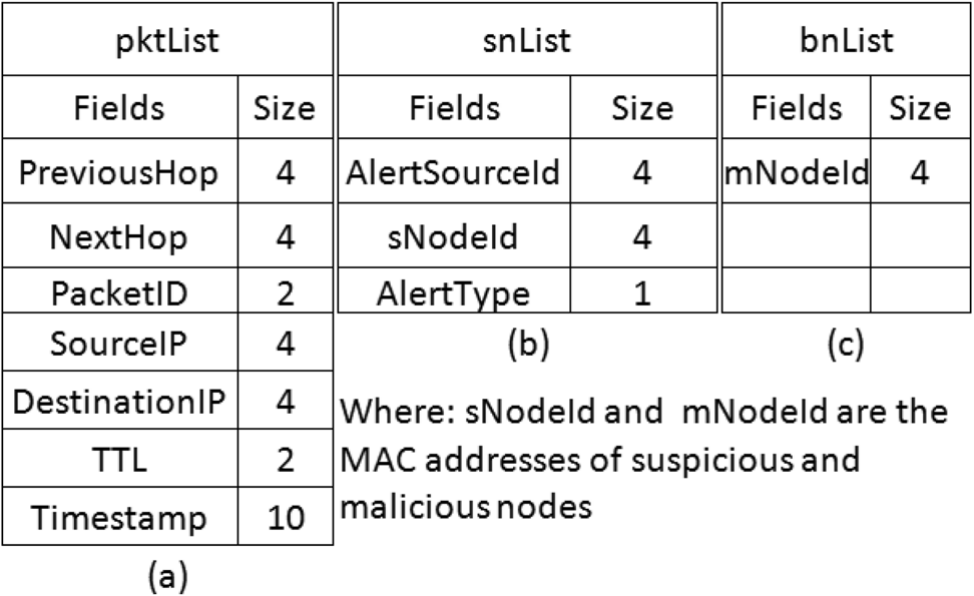
DAIWN Cache Lists.

The flexibility and versatility of the proposed approach make it extendable and scalable. It can be extended to mitigate other attacks as well such as jamming using network performance parameters as proposed by Carolina et. al.^77^. Moreover, it can be deployed to any size of the network. In the proposed approach, the packets are recorded in the PktList, and suspicious and malicious nodes are recorded in the snList and bnList respectively. Formats of these storage structures are given in Fig. 3. For overhead processing and enabling the reader to understand the detection algorithm, a filled-in data structure of pktList is given in Table 3.

**Table 3:** Entries in the PacketList.

Packet	Previous Hop $\rightarrow$ Next Hop	Source IP	Destination IP	TTL	Time Stamp
A	L(1)$\rightarrow$W(2)	192.168.1.1	192.168.1.6	5	2
A	W(2)$\rightarrow$W(10)	192.168.1.1	192.168.1.6	1	2
B	L(1)$\rightarrow$W(2)	192.168.1.1	192.168.1.6	5	1
B	W(2)$\rightarrow$W(10)	192.168.1.1	192.168.1.6	4	2
B	W(10)$\rightarrow$W(2)	192.168.1.1	192.168.1.6	3	3
C	L(1)$\rightarrow$W(2)	192.168.1.1	192.168.1.6	5	1
C	W(2)$\rightarrow$L(4)	192.168.1.1	192.168.1.6	32	2
C	L(4)$\rightarrow$L(5)	192.168.1.1	192.168.1.6	31	3
C	L(5)$\rightarrow$W(10)	192.168.1.1	192.168.1.6	30	4
C	W(10)$\rightarrow$L(5)	192.168.1.1	192.168.1.6	32	5
C	L(5)$\rightarrow$L(4)	192.168.1.1	192.168.1.6	30	6
C	L(4)$\rightarrow$W(2)	192.168.1.1	192.168.1.6	30	7
D	L(1)$\rightarrow$W(2)	192.168.1.1	192.168.1.6	5	1
D	W(2)$\rightarrow$L(4)	IP-W(2)	IP-W(10)	4	2
D	L(4)$\rightarrow$L(5)	IP-W(2)	IP-W(10)	3	3
D	L(5)$\rightarrow$W(10)	IP-W(2)	IP-W(10)	2	4
D	W(10)$\rightarrow$(5)	IP-W(10)	IP-W(2)	5	5
E	L(1)$\rightarrow$W(2)	192.168.1.1	192.168.1.6	5	1
E	W(2)$\rightarrow$W(10)	IP-W(2)	IP-W(10)	2	2
E	W(10)$\rightarrow$W(2)	IP-W(10)	IP-W(2)	1	3
F	L(1)$\rightarrow$W(2)	192.168.1.1	192.168.1.6	5	3
F	W(2)$\rightarrow$W(10)	192.168.1.1	192.168.1.6	4	1
F	W(2)$\rightarrow$W(10)	192.168.1.1	192.168.1.6	5	2
F	W(10)$\rightarrow$...	192.168.1.1	Broadcast-IP	4	3

**Algorithm 1:**
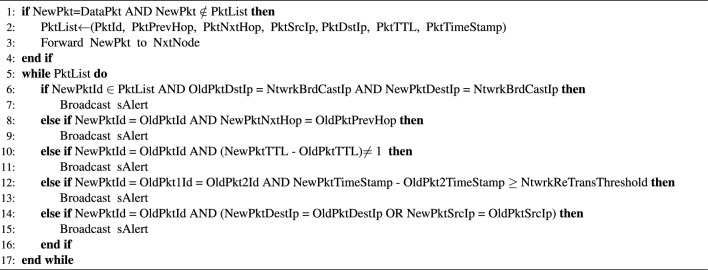
wormhole detection

### Isolation of malicious nodes

If a legal node receives sThreshold (n) number of sAlerts (suspicious alert), then it generates an mAlert (malicious alert) whose threshold is termed as k (refer Eqs. (1) and (2). So, when any legal node receives mAlerts equal to the decided threshold, then all the entries of a reported malicious node are removed from the routing table. Thus, a legal node whose count of mAlerts completes first also generates a RERR (Route Error Message). Source node, on receiving RERR, broadcasts a new RREQ (Route Request Message). Every node blocks the RREQ from the nodes listed in their bnList. The RREQ message safely reaches the destination. The destination sends RREP (Route Reply Message), which reaches the source safely because the routes are symmetric and bidirectional in AODV. In this way wormhole nodes are isolated and a secure route is established between the source and the destination.

**Figure 4:**
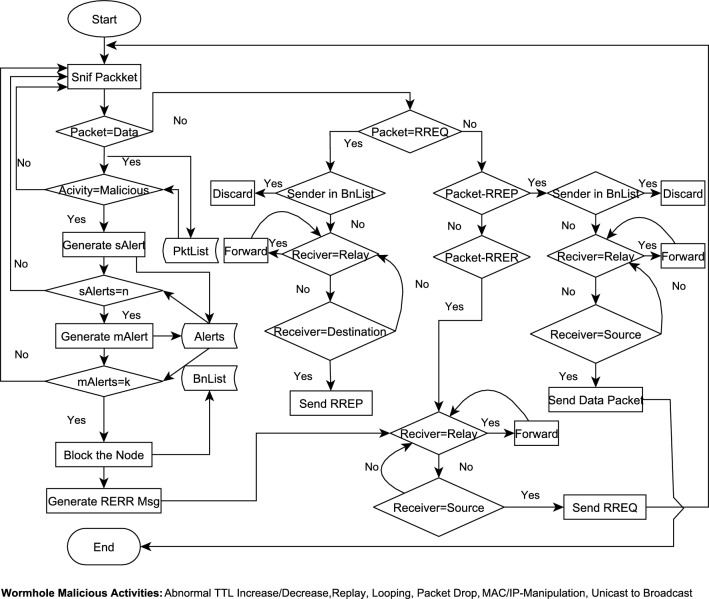
Detection and Isolation of Wormhole Nodes.

Information in Table 3 is the example of Pktlist maintained at each legitimate node. The information similar to this list is used for packet analysis to detect malicious behaviour of the attacker nodes. This data structure has seven columns namely; Packet-Id, Previous-Hop, Next-Hop, Source-IP, Destination-IP, TTL, and TimeStamp. This is the information which is used to detect malicious behaviour of the wormhole nodes. We have detected abnormal changes in TTL, IP-spoofing, replay, and flooding of the data packets as different malicious activities of the wormhole nodes. Time Stamp is stored using UNIX Time Stamp guidelines given in^78^. A few entries of the PktList are shown in Table 3 which gives the reflection of some malicious activities of different nodes. Each node maintains a snList and bnList. These data structures are used for the isolation of wormhole nodes. The formats of these storage lists are given in Fig. 3. The complete flow of detection and isolation of wormhole nodes is shown in Fig. 4 for easily comprehending the proposed solution.

Algorithm 2 is devised for the successful isolation of wormhole nodes.

**Algorithm 2:**
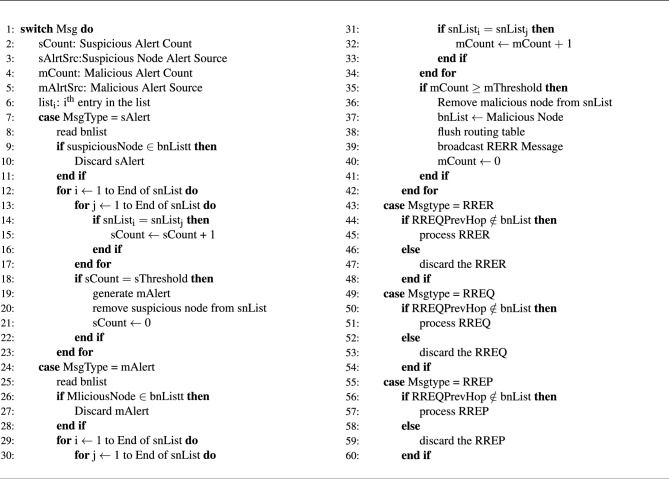
Wormhole Nodes Isolation

### Proof of concept

Proof of concept is tested via NS2.30 by simulating a wormhole attack using the scenario of wireless ad hoc networks IEEE 802.11 protocol. Two hundreds network nodes and 2-8 wormhole nodes are used for this purpose. The simulation setup used for the proof of concept is given in the Table 4. Purposefully, we have placed this section here because in the later sections some of these values are referred for estimation and calculation of the results.

**Table 4:** SIMULATION SETUP.

Properties	Value	Properties	Value
Channel Type	Wireless Channel	Normal Node Radio Range	80 m
Radio-Propagation Model	Two Ray Ground	Wormhole Radio Range	400 m
Antenna Model	Omni-Directional	Packet Size	512 Bytes
Protocol	AODV	Data Rate	64 Kb/Sec
Topology	1000 m x 1000 m	No. of Wormholes Nodes	2-8
Simulation	Time 5-100 Seconds	MAC	802.11
No. of Nodes	200	sAlerts (Suspicious Alerts)	5
Nodes Speed	0-5 m/Sec	mAlerts (Malicious Alerts)	1
Node Initial Energy	1000 Joules		

**Figure 5:**
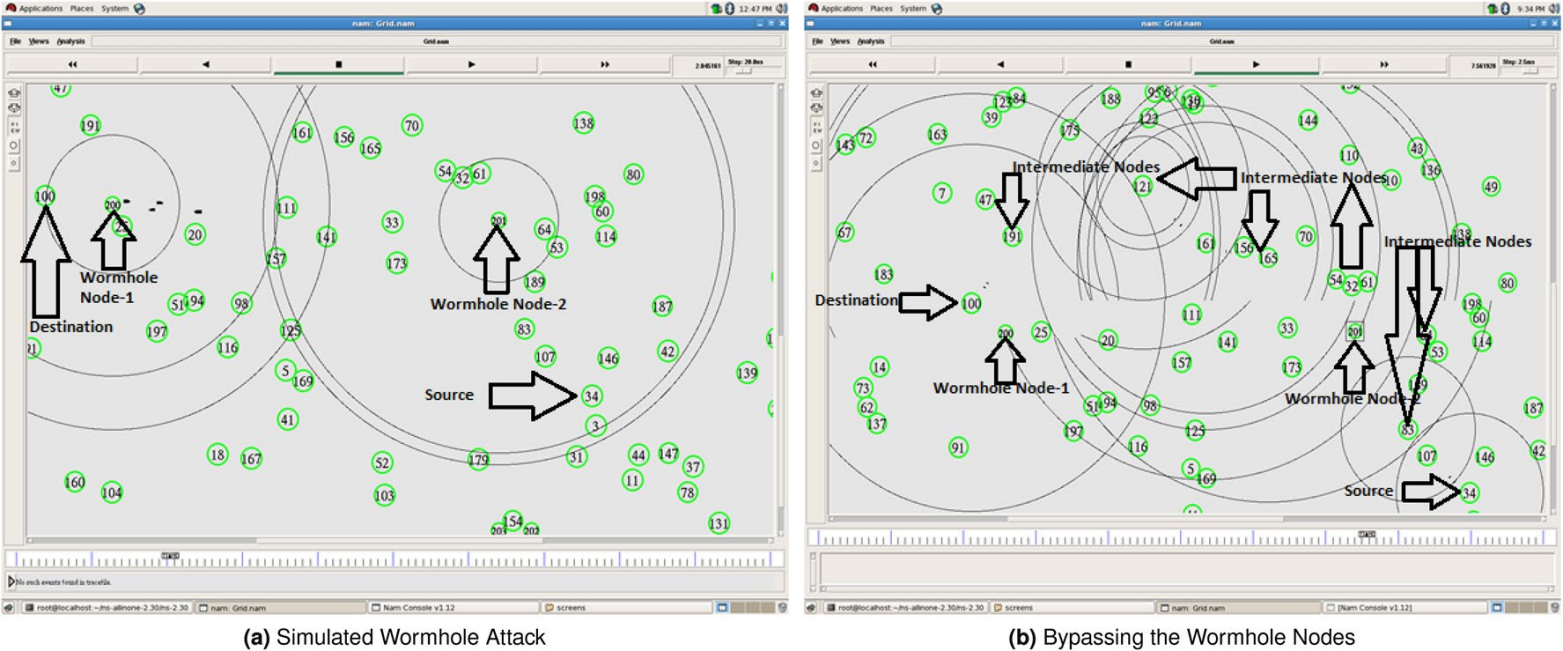
Simulation of Wormhole and its Mitigation.

In the scenario which is shown in Fig. 5, two high radio range ($400m^2$) wormhole nodes are strategically placed near the source and destination nodes. These are labeled as **200** and **201** in simulation scenario shown in Fig. 5a. These wormholes nodes successfully hijack the path between node-34 (source) and node-100 (destination) during route discovery process. We apply the proposed solution by modifying the same routing protocol (AODV), which is already proved susceptible to wormhole attack in above discussions. Our solution does not provide defence against the wormhole, rather it allows wormhole to occur, but then detects and isolates the colluding nodes based on their malacious actions. The colluding nodes are reported and placed in bnList (BlockList) and no RREQ packet is entertained from them by the legal nodes in the route discovery process later on. Figure 5b shows that legal nodes (source node-34 and destination node-100) successfully bypass the wormhole nodes and establish a communication channel through intermediate nodes (191,121,...,64,83).

## Detection time analysis

The tuning of parameters {**k, m**} also affects the detection and isolation times. Intuitively, wormhole detection time is a function of k, m, data size, and data rate. The detection time **Dt** can be derived using Eq. (4).

4 $$\begin{aligned} Dt = \left( k + m\right) *\left( \frac{Data~Size}{Data~Rate}\right) \end{aligned}$$

We would like to discuss that node distribution is a random event whose probability depends upon the number of nodes and area of the networks. Usually, it is modelled with Poisson Distribution as shown in Eq. (5).

5 $$\begin{aligned} P\left( N \right) = \frac{{e^{- \left[ \left( N-1\right) \frac{\pi R^2}{\Vert A\Vert }\right] \Vert A\Vert } \left[ \left[ \left( N-1\right) \frac{\pi R^2}{\Vert A\Vert }\right] \Vert A\Vert \right] ^N }}{{N!}} \end{aligned}$$

If put $\lambda =\left[ \left( N-1\right) \frac{\pi R^2}{\Vert A\Vert }\right]$ which is a node density factor, then the Eq. (5) can be written in more simplified form as $P\left( N \right) = \frac{{e^{-\lambda \Vert A\Vert } \left( \lambda \Vert A\Vert \right) ^N }}{{N!}}$. $\Vert A\Vert$ is an area of the network, and N is the number of nodes randomly distributed in this network. Node distribution is random, but we strategically deployed at least two wormhole nodes closer to the source and destination nodes as this attack mounts in reality. While looking into Table 5, we see the importance of setting the values of parameters k, m, data size, and data rate with great care. Here k and m are directly dependent on the factor $\left[ \left( N-1\right) \frac{\pi R^2}{\Vert A\Vert }\right]$ which cannot be ignored. It’s important to discuss that decreasing the number of alerts increases the detection time of wormhole nodes but decreases the accuracy of detection. It appears that the likelihood of detection increases with an increase in the number of nodes in the network, and decreases as the number of nodes decreases. Similarly, it seems that when the size of the network (measured in square meters) is expanded while keeping the number of nodes constant, the probability of detection decreases. This relationship is directly correlated with the radio range of the wireless nodes. However, the proposed detection and isolation mechanism is based on the 50% of neighboring nodes, which should report the malicious nature of the attacker. Keeping the network area constant and increasing the number of nodes will not significantly impact the detection time; the number of nodes required to meet the threshold of detection and isolation will increase, and vice versa. However, if the network becomes too dense, it may affect the detection time, as evident from the Table 5. In the case of decreasing the network area, the detection time slightly increased at A={200000, 100000}. The alert mechanism for detecting and isolating malicious nodes is not significantly impacted by these two factors in this case. However, if the percentage is ignored and some constant value is assumed, then these two factors significantly impact the detection and isolation mechanism. So, it is a matter of trade-off between detection time and accuracy. We leave it to the network designers to decide according to their requirements.

**Table 5:** Detection Time Analysis.

A ($m^2$)	N	R (m)	x	y	n	k	m	DR	DS	Dt for N=200, A=$10^6m^2$	Dt for N=200, A=$10^6m^2 to 10^5m^2$	Dt for N=200 to 110, A=$10^6m^2$
$10^3\times 10^3$	200	200	100	50	199	12.5	186.5	56	200	0.71	0.71,A=1000000	0.71,N=200
$10^3\times 10^3$	200	200	90	50	179.1	12.5	166.6	56	200	0.64	0.63,A=900000	0.63,N=190
$10^3\times 10^3$	200	200	80	50	159.2	12.5	146.7	56	200	0.57	0.56,A=800000	0.56,N=180
$10^3\times 10^3$	200	200	70	50	139.3	12.5	126.8	56	200	0.5	0.49A=700000	0.49,N=170
$10^3\times 10^3$	200	200	60	50	119.4	12.5	106.9	56	200	0.43	0.42,A=600000	0.41,N=160
$10^3\times 10^3$	200	200	50	50	99.5	12.5	87	56	200	0.35	0.35,A=500000	0.34,N=150
$10^3\times 10^3$	200	200	40	50	79.6	12.5	67.1	56	200	0.28	0.28,A=400000	0.27,N=140
$10^3\times 10^3$	200	200	30	50	59.7	12.5	47.2	56	200	0.21	0.21,A=300000	0.19,N=130
$10^3\times 10^3$	200	200	20	50	39.8	12.5	27.3	56	200	0.14	0.3,A=200000	0.12,N=120
$10^3\times 10^3$	200	200	10	50	19.9	12.5	7.4	56	200	0.07	0.82,A=100000	0.05,N=110
DR is Data Rate, DS is Data Size and DT is Detection Time; formulae used to calculate detection time, n, k, m are:												
$n = (N - 1) \times x/100$, $k = (N - 1) \times 1/100 \times y\pi R^2 / A$, $m = (N - 1) \times 1/100 \times (x - y\pi R^2/A)$, $DT = (k + m) \times (DS/DR)$												

**Figure 6:**
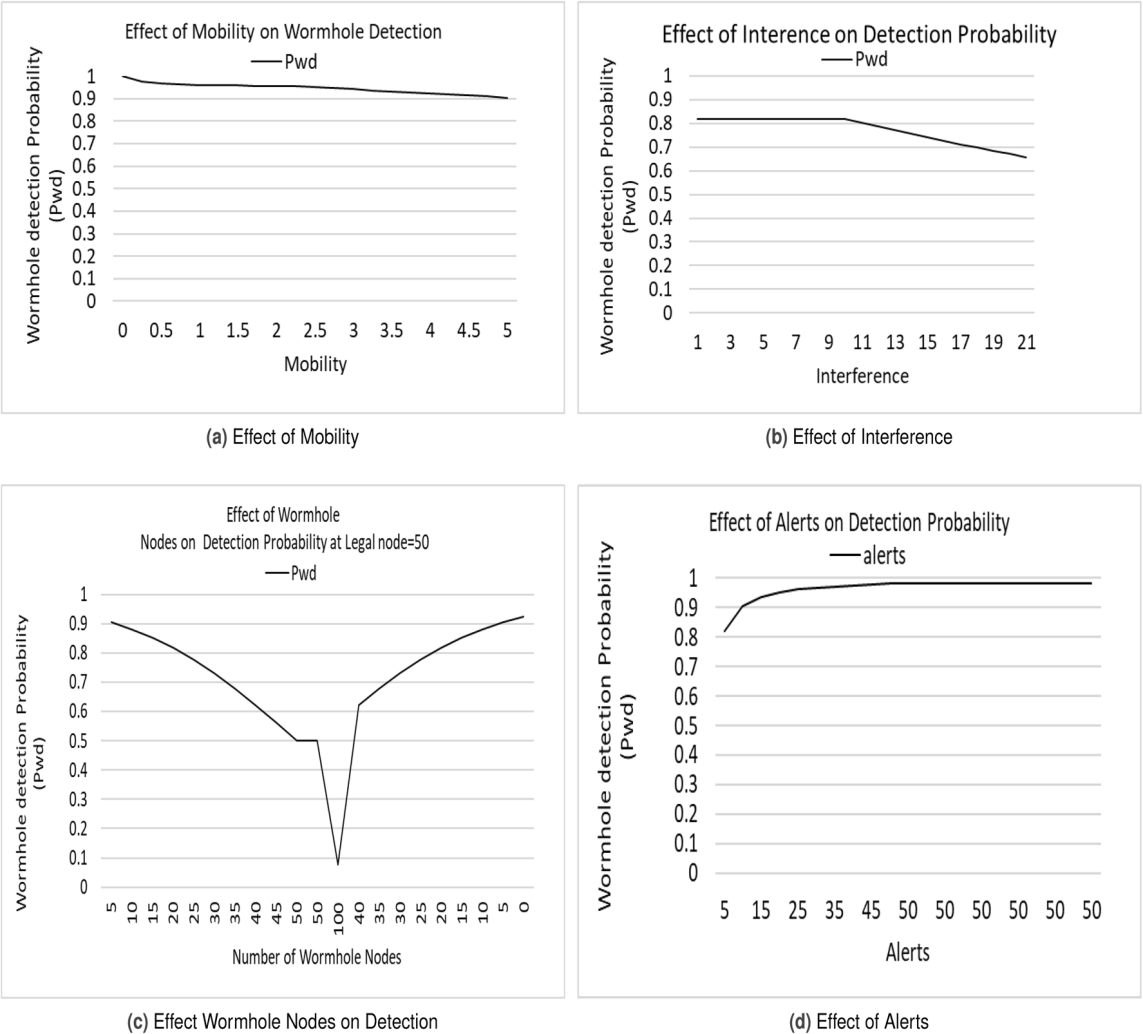
Effect of Interference, Mobility, Malicious Nodes and Alerts on Wormhole Detection.

The relationship between wormhole detection probability, the number of legitimate and malicious nodes, interference (in terms of frame losses), and mobility is illustrated in Fig. 6. The figure indicates that the detection probability rises with the number of legitimate nodes. However, when the number of malicious nodes becomes equal to or surpasses the number of legitimate nodes, the probability starts to decrease. This behavior is captured by a sigmoid function, as presented in Eq. (6), and visualized in Fig. 6d. The detection probability remains at zero if no alerts are generated, and it increases with the growing number of alerts, and vice versa.

6 $$\begin{aligned} P(D | N, W) = \frac{1}{1 + exp^{-k * (N - W)}} \end{aligned}$$

In the case of interference, the detection process is adversely affected due to communication disruption, resulting in a high frame loss rate. Consequently, both the generation and reception of alerts are diminished, leading to a reduction in the detection probability. This phenomenon is illustrated in Fig. 6b. A similar scenario occurs with mobility, where the frequent creation and breakage of routes lead to reduced throughput. This, in turn, affects the reporting of malicious nodes and compromises the detection probability. This effect is depicted in Fig. 6a.

To be honest, natural interference is relative in nature, impacting the efficiency of both legitimate and malicious nodes. However, artificial interference requires careful consideration. Equation (7) is defined to model the effects of interference, mobility, malicious nodes, and alerts on detection. The individual impacts of these parameters are demonstrated in Fig. 6c.

7 $$\begin{aligned} P(WD)= \left\{ \begin{array}{ll} 0 &{} if ~ number~ of ~alerts~ A=0 \\ (1-\frac{1}{N*A})^{N*I*M*W} &{} otherwise,~provided~N>=W \end{array} \right. \end{aligned}$$

In this equation

N: number of legal nodes

W: number of malicious nodes

I: Interference

M: Mobility

A: Alerts

## Overhead analysis

We believe a fair cost-and-benefit analysis of security solutions helps the research community and industry to evaluate and improve them in a more meaningful way. That’s why we have provided a detailed analysis of overheads and the costs connected with the proposed solution. It became difficult for us to decide what reference values should be used for cost and benefit analysis. After studying the published material, we came across one of the articles by Moslem Amiri^79^ that gives some values, that can be used as reference points for a relative estimation of energy consumption. To calculate read/write and messaging overheads, these values are given in Table 6. All energy values are represented using Joule(J). Before moving towards the calculation of overheads, we would like to refer to the data structures that are used by our approach; refer to Fig. 3.

**Table 6:** Reference Points for the Calculation of Overheads.

Sr.	Operation	Energy Used
1	write 1 byte	0.0349 mJ
2	send 1 bit	15 $\mu$J
3	read 1 byte	0.0082 mJ
4	run 1 instruction	0.2 nJ

### Storage overhead

In this section, we calculate the storage overhead of our proposed approach heuristically. For the sake of derivations simplicity, we have represented a number of items in PktList, SnList and BnList as X, Y, Z and we represent records in these lists as RX, RY and RZ. Thus, the total storage overhead in terms of a number of bytes can been expressed using Eq. (8).

8 $$\begin{aligned} Mem_{Bytes}=\sum _{i=1}^{X}RX_i+\sum _{j=1}^{Y}RY_j+\sum _{k=1}^{Z}RZ_k \end{aligned}$$

If $Mem_{Bytes}$ is the total memory bytes, then the energy consumption to process (Read/Write) $Mem_{Bytes}$ these bytes can be expressed using the following Eq. (9).

9 $$\begin{aligned} Mem_{Energy} =Mem_{Bytes} * \left( \frac{\alpha +\beta }{2}\right) \end{aligned}$$

Assuming Area=1000m x 1000m, Nodes=200, $R_n$(radio range of normal node)=200, $R_w$(radio range of wormhole)=400; then *k* would be 12.49. If there are 10 mNodes (malicious) and single pktListEntry=32Bytes, snListEntry=9Bytes, bnListEntry=4, and an average number of entries to detect malicious node in the pktList=4, then a single node can store the activities of $(N-1)*\pi *R_n^2/A$ nodes. In this way, the size of pktList becomes $32*(N-1)*\pi *R_n^2/A$ i.e 803.34 Bytes and the size of snList becomes $9*(N-1)*\pi *R_w^2/A$ i.e 450 bytes. Size of bnList depends upon the number of malicious nodes detected truly and legal nodes detected falsely. In this scenario, it would cost about 40 bytes for 10 malicious nodes. Finally, $Mem_{Bytes}$ is 803.34+450+40=1293.34Bytes, and, $Mem_{Energy}$ is 0.02155*1293.34=27.87mJ.

### Messaging overhead

The transmission of messages in a wireless network consumes a considerable amount of power. The power consumption of one-byte message transmission is about 80 times greater than the power consumption of one-byte read/write operation according to Table 6. So, it is a critical factor which is less explored by the research community. But we have brought it into the discussion in order to improve the manuscript. Each of malicious and suspicious node-id is of 4-bytes. For broadcasting, these node ids, and malicious and suspicious alert messages are used, respectively. The messaging overhead in terms of a number of bytes can be obtained from Eq. (10). Similarly, the power consumed for transmitting these alerts in terms of energy (Joules) can be estimated using Eq. (11).

10 $$\begin{aligned}{} & {} Msg_{Bytes}=\sum _{i=1}^{m}mAlert_i+\sum _{j=1}^{k}sAlert_j \end{aligned}$$

11 $$\begin{aligned}{} & {} Msg_{Energy}=\gamma *Msg_{Bytes} \end{aligned}$$

So, in this case, $Msg_{Energy}$ would be $15*32=480$ $\mu$J, which is proportional to an active number of attackers in the networks. Our proposed solution is bandwidth effective, since, its message size is 4-Byte which is quite effective as compared to other techniques which exchange complete routing table or neighbour list e.g^66^. Moreover, alerts are generated only when suspicious or malicious activity is encountered.

**Figure 7:**
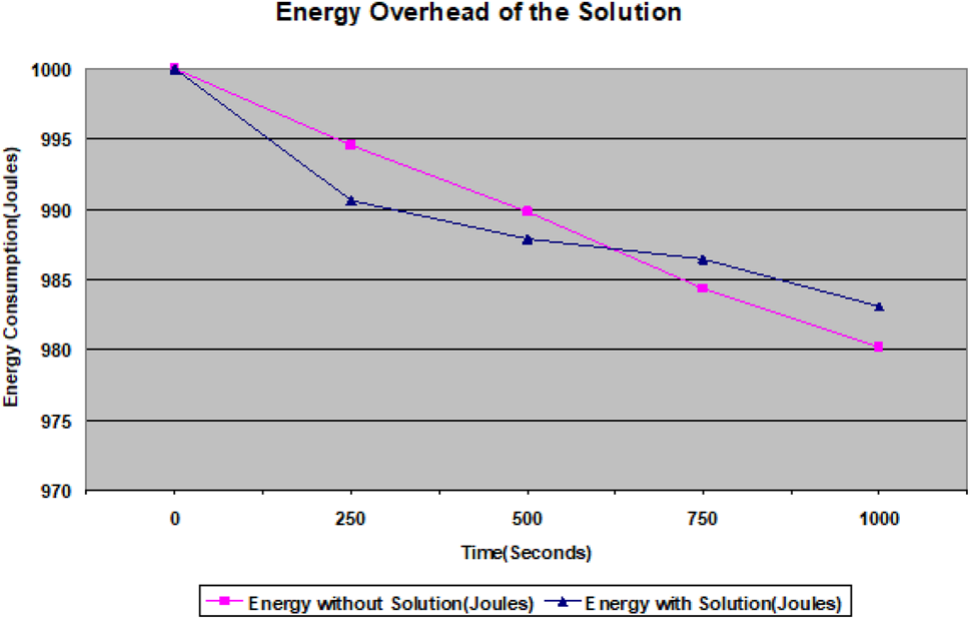
Energy Consumption.

From the simulation, it is observed that the proposed solution consumes a very small amount of energy for the detection and isolation of wormhole nodes. Figure 7 shows a minute gap between two lines of energy consumption in AODV without and with a solution. The network utilizes more energy in the absence of a solution because of re-transmissions carried out due to frames dropping or looping as a result of the malicious behaviour of wormhole nodes. Figure 7 shows 1000 seconds simulation in which energy consumption is just about 17 Joules whereas in the absence of a solution it is about 20 Joules. In addition, at about 625s, the proposed solution consumes more energy due to the extra transmission of alert frames as compared to normal AODV, that does not do this. After that energy consumption becomes stable. Equation (12) helps calculate average energy consumption.

12 $$\begin{aligned} E_{Avg}=\frac{1}{N} \sum _{k=1}^{N} \left( E_ik-E_fk\right) \end{aligned}$$

In the above equation, $E_{Avg}$ is average, $E_ik$, and $E_fk$ are initial and final energy values for node i. N is the total number of nodes in the network.

**Figure 8:**
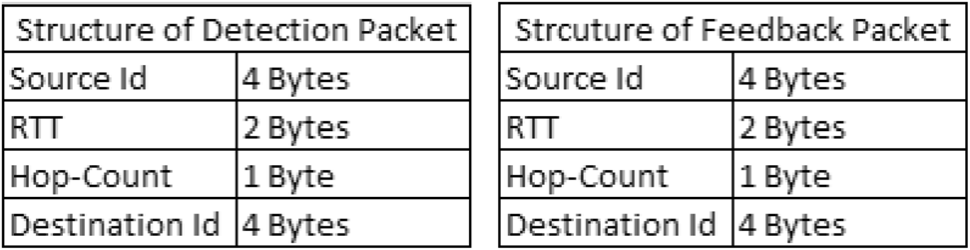
Structure of DP and FP Packets.

Moreover, in 100 seconds simulation, the energy consumed by our solution is less than DWASPS^49^ which consumes more than 20 Joules, whereas our solutions consume about 8-10 Joules. This clearly indicates the efficacy of the proposed solution for the longer life of the network. In our case, energy consumption is partially dependent on the number of attackers. It only depends on the active ones, meaning those who are performing malicious activities. The high cost of energy consumption of DWASPS^49^ is also due to the use of Particle Swarm Optimization (PSO). In addition to this DWASPS^49^ high energy consumption is also due to the use of two packets i.e. Detection-Packet (DP) and Feedback Packet (FP). These are larger in size than our alert messages. Their structure and size is shown in Fig. 8.

**Figure 9:**
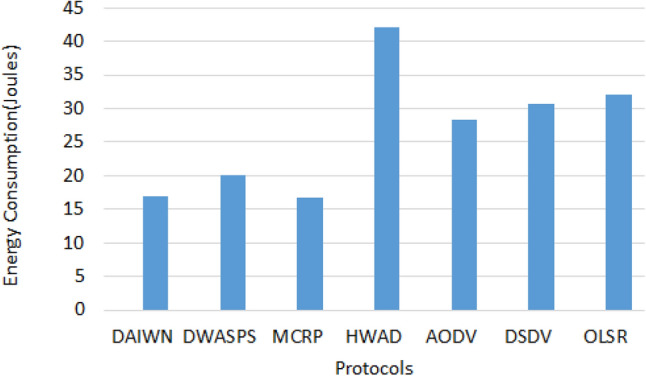
Energy Consumption Comparison.

It is observed that our proposed solution performs better than MCRP^50^ and DWASPS^49^ in terms of energy consumption. The energy consumption in the case of MCRP^50^ is 16.66J whereas in our case, it is 17J. But our solution outperforms MCRP^50^ because 16.66J is the energy consumption of the static scenario and no results of the mobile scenario are reported. Our solution is a an improvement in the original MANETs routing protocols under attack, such as AODV, DSDV, or OLSR. The comparison with different protocols is shown in Fig. 9.

### Computation overhead

The proposed approach uses linear lists to detect and isolate wormhole nodes. It incurs a computation cost of *O(n)*, which is equal to the computational cost incurred by HWAD^46^. But our solution outperforms DWASPS^49^ whose computational cost is $O(n^2)$ which in turn depends on PSO with the computational complexity of $O(n^2)$ while calculating Euclidean distance between particles used to form the neighbour list^80^.

The computation required for these lists (pktList, bnList and snList) is also small. For example, a MICA mote with ATMEGA-128 4-MHz processor takes about 2 microseconds to look up a buffer of 100 entries. The time to keep packets in the pktList and snList is also very small that is $\approxeq$ 0.15 seconds which is calculated in ns-2 installed at RedHat-5 enterprise edition. But this duration will be much less in a real hardware-based environment as opposed to a software-based simulation. The entries from bnList are not removed as they help to defend the power of the system. Since the computational cost is negligible, that’s why the proposed solution is suitable for mitigating wormhole attacks in resource-constrained wireless scenarios.

### Packet loss

Because the packet loss ratio is one of the important factors that is used to measure the efficiency of any communication system. The basic equation that was used to calculate packet loss ratio is given by Eq. (14) which is extended from Eq. (13).

13 $$\begin{aligned}{} & {} Packet~Delivery~Ratio=\left[ 100*\left( \frac{Data~Recieved}{Data~Sent}\right) \right] \end{aligned}$$

14 $$\begin{aligned} Packet~Loss~Ratio=100-\left[ 100*\left( \frac{Data~Recieved}{Data~Sent}\right) \right] \end{aligned}$$

Where $\{100*Data~Recieved/Data~Sent\}$ is the delivery ratio. The packet loss ratio with 2, 4, 6, and 8 wormhole nodes is acquired from the SN2 simulation. DAIWN packet loss rate is the average value. It is an output of a simulation that was run 10 times with 2,4,6,8 wormhole nodes. We recorded the traffic in the gridw2move.tr, gridw4move.tr, gridw6move4.tr and gridw8move.tr tracefiles.

**Figure 10:**
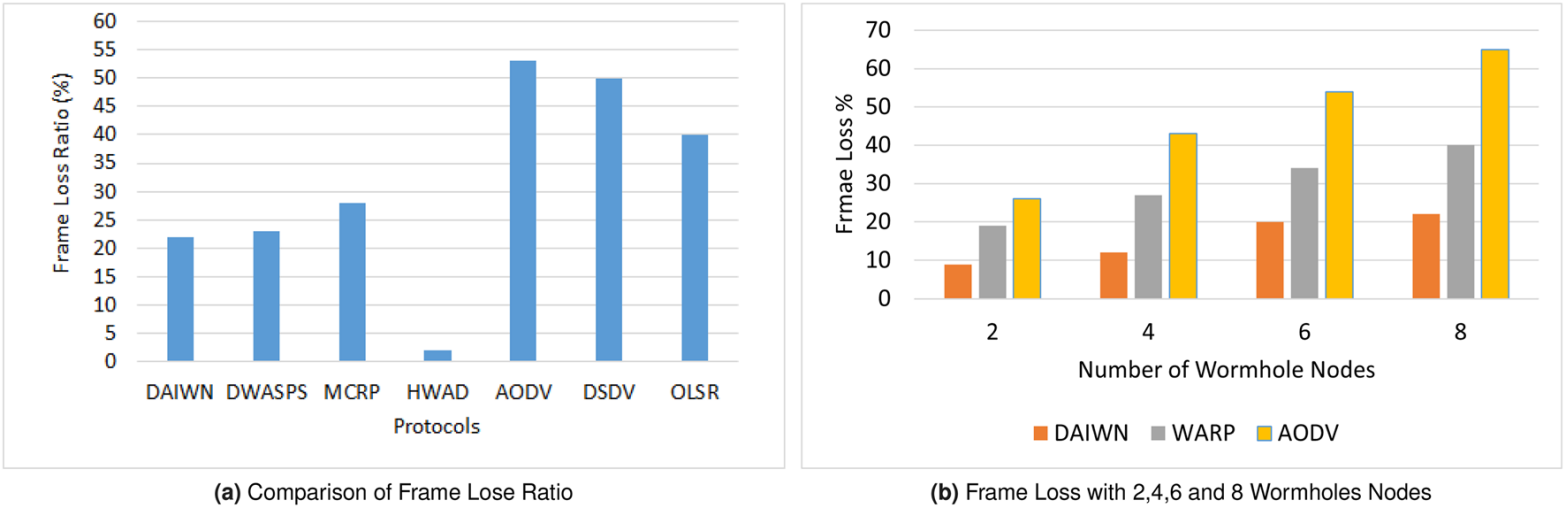
Frame Loss.

The trace files were passed to a Pearl script for the calculation of packet loss and delivery ratio. This script can be found at^81^. The single simulation showed quite high values of packet delivery and low value of frame loss ratios, which on average were 84% and 16% respectively in the mobile scenario. Whereas, for 10 rounds, the average values were found to be 78% and 22% respectively. It is observed that, the packet loss rate increases with the mobility of wireless nodes. With respect to the delivery of packets, in the static scenario our solution performs better than DWASPS^49^ by $\approx$ 1%. But its performance deteriorates in mobile scenario due to mobility. The results of mobile scenarios are not published by DWASPS^49^, that is why, we cannot compare our results of mobile scenarios with DWASPS^49^. Moreover, the proposed solution performs better than most of the approaches such as MCRP^50^ and brings an improvement in basic reactive routing protocols like AODV, DSDV and OLSR that poorly perform under attack scenarios. Figure 10a shows that HWAD^46^ has small frame loss ratio, but if we look at Fig. 9, then we can see that it has the highest energy consumption rate which is hard to justify in low power devices.

Researchers might be interested in knowing the individual frame losses in cases 2, 4, 6 and 8 wormhole nodes. We could find only a single article by Ming-Yang Su named WARP^52^ that finds frame loss in scenarios with 2,4 and 8 wormhole nodes. The missing frame loss value is determined by using linear interpolation^82^. Frame loss with the above mentioned number of wormholes is shown in Fig. 10b. It shows that DAIWN has lower losses in all scenarios as compared with WARP and AODV.

### Statistical analysis of detection rate

In secure communication systems, it is very important to measure the effectiveness of the security model. Once the model is built, the next task is to find the fitness of the model. That is why, we evaluated the performance of our proposed solution in this section through accuracy, the F1 score and Matthews correlation coefficient ($M_{cc}$). The values of these tests are calculated using the Eqs. (15), (16), (17) and the results are given in Table 7.

*(i) Accuracy: * It is a simple metric which is the ratio of correctly detecting the malicious links with respect to the total detection/observations. Equation (15) is used to calculate the accuracy.

15 $$\begin{aligned} Accuracy=\frac{TP + TN}{TP + TN + FP + FN} \end{aligned}$$

The proposed solution has a high accuracy of wormhole detection. On average, it has 99%. But in statistical analysis accuracy may lead to over-optimism. That’s why other parameters are also evaluated especially F1-Score and $M_{cc}$.

*(ii) F1 Score: * It is the geometric mean of precision and recall and it lies between them. Equation (16) is used to calculate it.

16 $$\begin{aligned} F1~Score=2*\frac{Recall * Precision}{Recall + Precision} \end{aligned}$$

Where *Precision* is a ratio of correctly observed wormhole links with respect to the total positive observation and is calculated as $Precision=\frac{TP}{TP+FP}$.

A recall is also called sensitivity. It is a ratio of correctly detected wormhole links with respect to all the wormhole links that actually exist. It can be calculated by $Recall=\frac{TP}{TP + FN}$. F1-Score greater than 0.50 is considered to be a good score that affirms the viability of a detection model. In our case, on average, the value of F1-Score is 0.68 which affirms the authenticity of our proposed model.

*(iii) Matthews correlation coefficient *($M_{cc}$): ($M_{cc}$) is a more reliable statistical test that gives results between -1 and +1. The value of this parameter is high if the model determines the values of categories and the confusion matrix efficiently. $M_{cc}$ result greater than zero and close to 1 indicates that the model is good. Equation (17) is used to calculate the value of $M_{cc}$. In our case, 0.73 is the average value, which indicates the usefulness and authenticity of our model in terms of attack detection and isolation.

17 $$\begin{aligned} M_{cc} = \frac{TP * TN + FP * FN}{\sqrt{(TP+FP) * (TP+FN) * (TN+FP) * (TN+FN)}} \end{aligned}$$

**Table 7:** Performance.

Case-1: High Radio Range Attacker													
WN	LN	WL	LL	LL DW	FN	TN	FP	TP	Accuracy	Precision	ReCall	F1 Score	Mcc
2	200	1	1225	2	0	1223	2	1	1.00	0.33	1.00	0.50	0.58
4	200	6	1225	4	0	1221	4	6	1.00	0.60	1.00	0.75	0.77
6	200	15	1225	0	0	1225	0	15	1.00	1.00	1.00	1.00	1.00
8	200	28	1225	0	0	1225	0	28	1.00	1.00	1.00	1.00	1.00
Case-2: Low Radio Range Attacker													
WN	LN	WL	LL	LL DW	FN	TN	FP	TP	Accuracy	Precision	ReCall	F1 Score	Mcc
2	200	1	1225	6	0	1219	6	1	1.00	0.14	1.00	0.25	0.38
4	200	6	1225	15	0	1210	15	6	0.99	0.29	1.00	0.44	0.53
6	200	15	1225	14	0	1211	14	15	0.99	0.52	1.00	0.68	0.72
8	200	28	1225	12	0	1213	12	28	0.99	0.70	1.00	0.82	0.83
On Average									0.99	0.57	1	0.68	0.73

**Figure 11:**
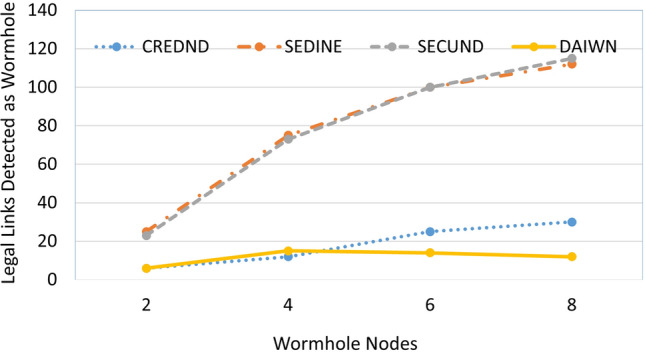
Comparison of Legal Links Detection as Wormhole Nodes.

Our proposed approach outperforms in terms of wormhole detection as the statistical scores of all parameters (Accuracy, Precision, Re-Call, F1-Score and MCC) are above the threshold value of 0.50. It is observed that it performs very well in case-1 where there are high radio range attackers, but its performance deteriorates in case-2 when there are low radio range attackers. This is due to the limited radio range of attackers that they use to exploit the legal nodes and thus compromise them to achieve malicious objectives. But, in the case of high radio range, they do not need to do that or they need to exploit a negligible number of legal nodes for accomplishing wormhole links. The detection rate of our approach is 98-99% by using equation $Detection~Rate=100-(FP \%+FN \%)$. We have also compared our results with other techniques namely; {CREDND^53^, SECUND^83^ and SEDINE^84^}. We observed that our proposed solution outperforms CREDND^53^, and SECUND^83^ as shown in Fig. 11. It is also observed that in the case of four wormhole nodes, our solution generates more false alerts. It is because of the wormhole nodes that are strategically placed to disturb the maximum traffic and thus compromise a greater number of legal nodes. It eventually results in a larger number of false alerts. Similarly, two wormhole nodes have lesser chances of collusion as compared to six and eight wormhole nodes. If we have more wormhole nodes, then they have a lesser dependency on legal nodes, which can consequently increase the chances of establishing a malicious link.

The theoretical time to detect a wormhole node lies between 0.07-0.71 seconds. But, from the simulation, it is found that our solution detects a wormhole node, isolates it and recovers the network from the attack within 0.151–1.186 seconds. So, on average, it is 0.67 seconds which 16% improvement in the detection time as compared with E2SIW.

## Limitations and research challenges

We believe, besides all the convincing aspects of any research, there is always room for improvement. That’s why it is the primary responsibility of the researcher to reveal the limitations or potential challenges of their research work. This attitude streamlines the efforts that are made towards the maturity and refinement of this particular aspect of research. So, keeping in consideration the improved perspectives of this study, we honestly disclose that our proposed solution isolates compromised nodes between two ends of the wormhole tunnel. Furthermore, if the number of attackers becomes greater than legitimate nodes, it can alter the isolation mechanism knocking out the legitimate nodes from the network. These are the open challenges that requires the research community to create a solution which can reduce the number of compromised nodes and improve the isolation process. The second challenge is to see, how we can use the long-range capability (fast wormhole tunnel) for the transmission of users’ legal data. Moreover, proposed technique cannot fight with attacker who performs a malicious action and then change its position and id.

## Conclusion and future work

This is a low-cost solution that achieves 98-99% detection with a frame loss rate of 22% in mobile scenarios without using additional hardware, time synchronization and complex cryptographic functions. Our proposed solution detects wormhole nodes, isolates them and recovers the communication system from infection within 0.151 seconds and in the worst case, it is 1.186 seconds. On average it is 0.67 seconds. The computation complexity of the detection and isolation algorithm is linear *O*(*n*) complexity. The proposed solution uses a small message size that is just 4 bytes and thus uses a small storage space of about 1.26 KB. This proposed solution does not impose any special requirement on the network and has small memory and messaging costs, that’s why it is highly suitable for resource-constrained systems. Our future work is to avoid or at least minimize the number of compromised nodes that are being isolated along with malicious nodes by our solution. This is the only limitation; otherwise, the solution is compatible with the existing network stack. The proposed solution can be easily incorporated at the routing layer in the form of software and no hardware changes are required at lower layers.

In the future, we plan to enhance and assess this methodology for minimizing the risk of isolating compromised nodes through the isolation algorithm. Additionally, we aim to prevent attackers from eliminating legitimate, non-compromised nodes when their count surpasses that of legitimate nodes.

## Data Availability

The datasets used and/or analysed during the current study available from the corresponding author on reasonable request.
